# An Investigation Into Physical Frailty as a Link Between the Gut Microbiome and Cognitive Health

**DOI:** 10.3389/fnagi.2018.00398

**Published:** 2018-12-04

**Authors:** Serena Verdi, Matthew A. Jackson, Michelle Beaumont, Ruth C. E. Bowyer, Jordana T. Bell, Tim D. Spector, Claire J. Steves

**Affiliations:** ^1^Department of Twin Research and Genetic Epidemiology, King’s College London, London, United Kingdom; ^2^Neuropsychopharmacology Unit, Centre for Psychiatry, Division of Brain Sciences, Imperial College London, London, United Kingdom; ^3^Kennedy Institute of Rheumatology, University of Oxford, Oxford, United Kingdom; ^4^Clinical Age Research Unit, Department of Clinical Gerontology, King’s College Hospital, NHS Foundation Trust, London, United Kingdom

**Keywords:** cognition, microbiome, twins, frailty, CANTAB, reaction-time, verbal-reasoning, MMSE

## Abstract

The preservation of cognitive abilities with aging is a priority both for individuals and nations given the aging populations of many countries. Recently the gut microbiome has been identified as a new territory to explore in relation to cognition. Experiments using rodents have identified a link between the gut microbiome and cognitive function, particularly that low microbial diversity leads to poor cognition function. Similar studies in humans could identify novel targets to encourage healthy cognition in an aging population. Here, we investigate the association of gut microbiota and cognitive function in a human cohort considering the influence of physical frailty. We analyzed 16S rRNA gene sequence data, derived from fecal samples obtained from 1,551 individuals over the age of 40. Cognitive data was collected using four cognitive tests: verbal fluency (*n* = 1,368), Deary-Liewald Reaction Time Test (DLRT; *n* = 873), Mini Mental State Examination (recall; *n* = 1,374) and Paired Associates Learning from the Cambridge Neuropsychological Test Automated Battery (CANTAB-PAL; *n* = 405). We use mixed effects models to identify associations with alpha diversity, operational taxonomic units (OTUs) and taxa and performed further analyses adjusting for physical frailty. We then repeated the analyses in a subset of individuals with dietary data, also excluding those using medications shown to influence gut microbiome composition. DLRT and verbal fluency were negatively associated with alpha diversity of the gut microbiota (False-Discovery Rate, FDR, *p* < 0.05). However, when considering frailty as a covariate, only associations between the DLRT and diversity measures remained. Repeating analyses excluding Proton pump inhibitor (PPI) and antibiotic users and accounting for diet, we similarly observe significant negative associations between the DLRT and alpha diversity measures and a further negative association between DLRT and the abundance of the order Burkholderiales that remains significant after adjusting for host frailty. This highlights the importance of considering concurrent differences in physical health in studies of cognitive performance and suggests that physical health has a relatively larger association with the gut microbiome. However, the frailty independent cognitive-gut microbiota associations that were observed might represent important targets for further research, with potential for use in diagnostic surveillance in cognitive aging and interventions to improve vitality.

## Introduction

Cognitive dysfunction in an aging population is a rising clinical and social concern, which has been described as “the elephant in the room” (Brayne, 2007). Cognitive traits particularly vulnerable to the effects of age are so-called “fluid” abilities such as fluency, speed of processing and memory. Changes occur across the population and are not confined to individuals diagnosed as having dementia (Albert et al., 1995; Chyou et al., 1996; Comijs et al., 2004; Schaie et al., 2004; Baltes et al., 2006). A small shift in the population’s overall trajectory of cognitive aging would potentially have effects at many levels—including reducing numbers crossing the threshold to dementia.

The assessment of frailty has shown to improve the clinical and research approach to cognitive disorders (Canevelli et al., 2017; Yu et al., 2018). Frailty is a condition characterized by a diminishment of an individual’s homeostatic reserves, leading to an increased vulnerability to exogenous and endogenous stressors (Coelho et al., 2015). Studies have shown gait speed and physical manifestations of the frailty syndrome to have a strong association with cognitive decline and dementia (Nyunt et al., 2017). For example, significant weight loss has shown to precede the onset of Alzheimer’s disease (AD; Cova et al., 2016). Poor grip strength, and diminished motor performance are associated with cognitive decline and a lessened risk of mild cognitive impairment (MCI), and conversion of MCI to AD (Kluger et al., 1997; Auyeung et al., 2008; Coelho et al., 2015). Low levels of physical activity are also associated with cognitive impairment (Searle and Rockwood, 2015; Steves et al., 2016). Despite its clinical relevance, the concept of physical frailty (weight loss, exhaustion, weaknesses, low gait speed and low physical activity) is rarely adopted in neurological settings and, in particular, in the field of cognitive disorders, which instead tends to focus on purely cognitive frailty as reduced cognitive reserve (Woods et al., 2013; Khezrian et al., 2017).

There is an urgent need to disentangle the complex network of pathophysiological mechanisms potentially affecting both cognition and individual vulnerability and resilience.

Amounting evidence links prolonged inflammation to cognitive disorders and dementia risk (Sartori et al., 2012; Vida et al., 2018). In older women, strength training has shown to decrease levels inflammation and to further improve cognitive scores (Chupel et al., 2017). Increasing and persistent levels of inflammation are associated with neurodegeneration and impaired neurogenesis (Delaby et al., 2015). Over the last few years, it has become widely accepted that the gut microbiome, the collective genome of commensal organisms in our gut, has important role in influencing inflammatory responses. The gut acts, not only as our largest interface with microbes and antigens, but also as the training ground for the immune system (Balzola et al., 2011; Levy and Borenstein, 2014). An imbalance to the structure of complex commensal communities in the gut microbiota has been postulated to increase pathogenic inflammatory mediators linked to range of health problems including cognitive health (Petersen and Round, 2014; Rogers et al., 2016).

Recent studies have made associations between gut microbiome and neurological disorders in animal models (Moos et al., 2016), particularly in rodents (Balzola et al., 2011; Bercik et al., 2011), including germ-free (GF) mice (Stilling et al., 2014). The gut-brain axis exists as a bidirectional relationship between the central and the enteric nervous system, linking together both emotional and cognitive functional areas of the brain with peripheral intestinal functions (Carabotti et al., 2015; Dinan and Cryan, 2017). Importantly, gut bacteria have been shown to alter host blood brain barrier integrity and thereby brain vulnerability to the influx of deleterious substances from circulation (Braniste et al., 2014). Diets high in saturated fat and added sugars target gut microbiota and alter endotoxin levels, which have been implicated in neuroinflammation, microglial activation and hippocampal vulnerability (Segain et al., 2000; Puig et al., 2012; Noble et al., 2017).

Animal models can only take us so far, as the gut microbiome is substantially different from species to species (Hugenholtz and de Vos, 2018). Some human studies have supported extrapolations from animal literature that the microbiome may be influential in human neurological processes. Altered diversity levels and a number of taxa have shown to be associated with autism (Finegold et al., 2002; De Angelis et al., 2013; Kang et al., 2013), multiple sclerosis (Chen et al., 2016; Jangi et al., 2016) and Parkinson’s disease (Scheperjans et al., 2015). These studies mostly compare small numbers of cases to healthy controls.

Very few studies have reported on association between the gastrointestinal microbiome and memory and cognition in adults. Recently, gut microbiota diversity, and some taxa were found to be reduced in 25 AD patients (characterized with Aβ_42_/Aβ_40_ CSF markers) compared to 94 healthy controls (Vogt et al., 2017). The common commensal* Faecalibacterium prausnitzii* positively associated and the *Enterobacteriaceae* family negatively associated with cognitive function in hepatic encephalopathy (Bajaj et al., 2012). In an elderly sample of dependent individuals, gut microbiome patterns associated with cognitive impairment measured using the Mini-Mental State Examination (MMSE; Claesson et al., 2012). However, all these studies may partly be confounded by overall health deficits (frailty), institutional dwelling, and altered diet in later stages of disease (Canevelli et al., 2017). In view of the substantial prodrome before dementia is realized, it is important that studies of the effect of the microbiome start early enough to establish the direction of any temporal association with cognitive decline. Moreover, the specific taxa identified have been similarly associated with frailty (van Tongeren et al., 2005; Claesson et al., 2012; Jackson et al., 2016c) and may have anti-inflammatory effect in rodents (Sokol et al., 2008; Miquel et al., 2015).

In an aging community dwelling cohort, we aimed to investigate the relationship between gut microbiome and cognitive ability. We hypothesized a reduction in microbiota diversity will be associated with poor cognitive function using cognitive domains associated with aging—speed, fluency and memory. We also hypothesized that this relationship between microbiota and cognition will be confounded by physical frailty.

## Materials and Methods

### Gut Microbiome Profiling

Fecal sampling, DNA extraction, and 16S rRNA gene sequencing was undertaken from *n* = 1,551 largely female (90%) members of the TwinsUK British twin cohort aged over 40 years (mean age 63, ranging 40–89), as part of an observational study of the human gut microbiome (Goodrich et al., 2016). Participants were unselected and community dwelling (Moayyeri et al., 2013; Steves et al., 2013). No patients had a current diagnosis of dementia. Metadata such as age and body mass index (BMI) were collected at the time of visit.

Chimeric sequences within the 16S rRNA gene sequencing data were removed using UCHIME (Edgar et al., 2011) and de novo sequences picked using Sumaclust in QIIME at a threshold of 97% (Jackson et al., 2016a), generating >1,000,000 operational taxonomic units (OTUs). Samples with less than 10,000 reads were excluded. Taxonomy was assigned to OTU representative sequences using UCLUST and the Greengenes 13_8 97% reference set. Counts were converted to relative abundance and log10 transformed following the addition of 0.000001 to account for 0 counts. OTU counts were also collapsed to taxonomic abundances between phylum and genus and similarly converted to relative abundances and log transformed as for OTUs.

The samples in this study were selected from a wider set of over 2000 samples, collection and storage methods are previously described (Goodrich et al., 2016). The OTUs and taxonomic summaries were subset to only those found in at least 50% of the samples in the study. These were selected for analysis as they are less sparse and hence more amenable to regression analysis (Goodrich et al., 2014). This resulted in 286 OTUs, 56 genera, 33 families, 16 orders, 13 classes and 8 phyla considered for analysis. Alpha diversity was calculated for each sample from the raw OTU tables using the alpha_diversity.py script in QIIME using four metrics, Shannon diversity, Chao1, Phylogenetic Diversity and number of unique OTUs.

### Cognitive Measures

To acknowledge the complexity and variation that occurs with cognitive traits, we used four different clinically validated measures of cognitive function: verbal Fluency Test, Deary-Liewald Reaction Time Test (DLRT) and Mini Mental State Examination (MMSE) and Cambridge Neuropsychological Test Automated Battery-Paired-Associated Learning Test (CANTAB-PAL). These cognitive data constitute all the cognitive measures collected during the routine TwinsUK cohort clinical visits between 2013 and 2016 and were matched to the nearest collected fecal sample.

#### Verbal Fluency

Processes involving language expression (such as naming, word finding and fluency) are a part of a normal and healthy neurocognitive operation (American Psychiatric Association, 2013). Verbal fluency is used in clinical evaluation to support diagnosis of cognitive impairment in persons with neurodegenerative diseases. Here, we assess verbal fluency in the TwinsUK cohort using the phonemic and semantic tests from the Addenbrookes Cognitive Examination III (ACEIII; Hsieh et al., 2013). The scoring used was the ACEIII total score out of 14. In models of the verbal fluency score, a reflected root transformation was used to account for negative skewing.

#### Deary-Liewald Reaction Time (Four Choice)

Reaction times capture the processing speed in decision-making and motor planning needed for higher-level cognitive functions (Wong et al., 2015). Such processes are subject to age-related cognitive decline. Subjects completed a computerized four choice reaction time task requiring subjects to make the appropriate response to one of four stimuli in the quickest time possible (Deary et al., 2011). The DLRT response time was normally distributed and required no transformation.

#### MMSE Recall

MMSE are designed to give a cross-sectional snapshot of a subject’s mental state in order to aid the diagnosis of dementia and other psychiatric disorders (Cockrell and Folstein, 1988). The MMSE is a 30-point questionnaire commonly used as a health screen that estimates the severity and progression of cognitive impairments and follows the course of cognitive changes in an individual (Tombaugh and McIntyre, 1992). Within this non-demented cohort, total MMSE was insufficiently variant, whereas part of the MMSE requiring the participant to recall three words after distraction, showed variance amenable to testing. We used this recall score (0–3) as a measure of memory function. Prior to modeling, the score was reflected root transformed as for verbal fluency, as it was similarly skewed.

#### CANTAB-PAL

The CANTAB includes sensitive, precise and objective measures of cognitive function that correlate to neural networks. We used the CANTAB-PAL test which assesses visual memory and new learning of shapes and location. Errors in learning the presentation of eight shapes in eight locations are counted and adjusted where the participant fails to complete the 8-shape task. This measure is of particular interest because of its ability to predict conversion from MCI to clinical dementia (Fowler et al., 1997; Blackwell et al., 2004), and predict cognitive decline (De Jager et al., 2005). For models we square root transformed the adjusted number of errors to account for its positive skew.

### Frailty

We used a 1–5 scale derived from the Fried phenotype which has five components: unintentional weight loss, self-reported exhaustion, weakness (grip strength), walking speed and physical activity, using standard methodology (Fried et al., 2001). Each subject was assigned a score ranging from 0 to 5 which we modeled as a continuous variable using the square root transformation due to positive skewing.

### Phenotype Associations With Covariates

Linear models were used to assess the significance of associations between each of the covariates with age, frailty and BMI. All statistical analysis was undertaken in R using the lm command (R Core Team, 2017). Variables were modeled as continuous with transformations. The direction of associations was visualized with fitted linear models using the geom_smooth command of ggplot2 within R.

### Association Analyses

Linear mixed effects models were used for all association analyses using the lme4 package in R (Bates et al., 2015). Models were run to investigate associations between microbiome variables at each level (alpha, OTUs and subsequent taxonomic levels). Microbiome traits were modeled as the dependent variable. Independent variables included the random effects of sequencing run, sample collection method (in person or by post), gender, family and relatedness (a variable encoded the same for MZ twin pairs and unique for DZ twins), sequencing depth was also included as a fixed effect to account for variability in read sampling between individuals. These models were also created including one of the four cognition variables as a fixed independent continuous variable. The models with and without the cognitive variable were then compared using the analysis of variance (ANOVA) function in R returning a *p*-value representing the significance of the difference between the nested models. Models were repeated to include the Fried frailty score as a covariate to account for physical frailty. These models (both with and without frailty) were carried out for all microbiome measures, for all four cognitive measures. All continuous fixed effects and dependent variables were scaled prior to modeling using the scale function. The reported *p*-values before and after frailty adjustment were corrected for multiple testing using the false-discovery rate (FDR) method (Benjamini and Hochberg, 1995) with the *p*.adjust command in R considering significance where corrected *p* < 0.05. Multiple testing corrections were made at the levels of microbiome measure type and phenotype, for instance adjusting for all the OTU level tests for verbal fluency.

### Medication and Dietary Effects

PPIs, antibiotics and diet are known to have a large effect on the gut microbiota and were well characterized within our cohort (Jakobsson et al., 2010; Wu et al., 2011; Jackson et al., 2016b). The cognitive outcome models were repeated considering these variables as potential confounders. PPI use was scored from self-reported prescription medication data taken from various questionnaires and considered positive if PPI use was reported at any point as described previously (Jackson et al., 2016b). Antibiotic use was scored based on self-reported use in the month prior to fecal sample collection. Dietary information was derived from food frequency questionnaire data (FFQ) collected following EPIC-Norfolk guidelines (EPIC-Norfolk Nutritional Methods: Food Frequency Questionnaire, 2014), and used to create the Healthy Eating Index (HEI; Guenther et al., 2013) and has been validated previously within this cohort (Bowyer et al., 2018). The data were subset to individuals with complete PPI, antibiotic and HEI data, further excluding individuals who reported taking PPIs or antibiotics. Modeling of cognitive measures was then repeated in this subset using the scaled HEI as a fixed effect to account for diet.

### Microbiome Associations With Physical Frailty

To contrast the relative breadth of microbiota associations with physical frailty as opposed to cognitive performance, we repeated the analysis with the Fried score in place of cognitive measures in turn. In this instance modeling used the full dataset (i.e., not subset by drug use and dietary data).

## Results

Sub-setting the TwinsUK gut microbiome dataset to individuals with complete data on age, BMI, Fried frailty, and at least one of the four cognitive measures considered (verbal fluency, DLRT, MMSE Recall, CANTAB-PAL; Table 1). Average scores were 12.14 on the verbal fluency (out of 14), 575 ms on the DLRT test, 2.58 in the MMSE recall test (out of 3, with a mean score of 29 across the whole MMSE), and 3.32 errors in the CANTAB-PAL test.

**Table 1:** Summary of study samples used for four cognitive measures.

Phenotype	Number	Age (Mean:SD)	BMI (Mean:SD)	Gender (M/F)
Verbal fluency	1,368	63:9	25.8:4.6	144/1,224
DLRT	873	62.5:8.9	25.8:4.5	89/784
MMSE recall	1,374	63:9.1	25.9:4.6	144/1,230
CANTAB PAL errors	405	63:7.9	26:4.69	37/368

**Figure 1:**
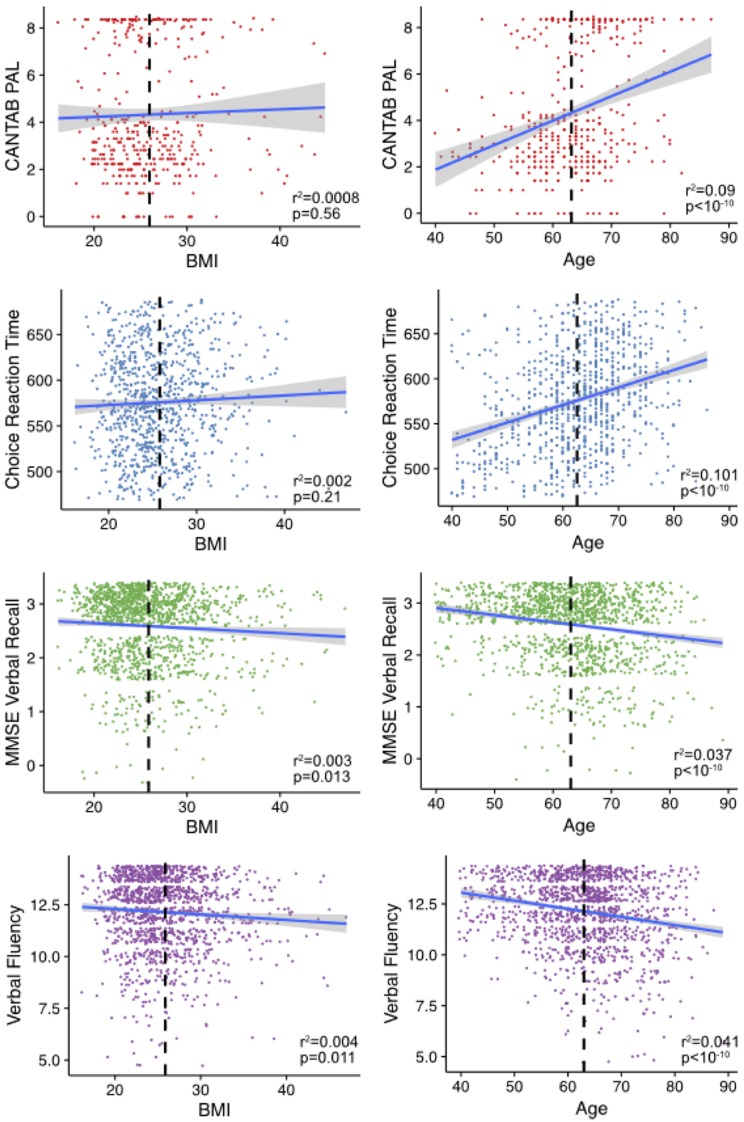
Age and body mass index (BMI) associations with cognitive measures. Shown are the age and BMIs plotted against the relative cognitive score for individuals in the study. Mean age and BMI in each of the sub-sets is shown in the dashed black line. The line is fitted using the geom_smooth command in ggplot2 within R. The variables verbal fluency and Mini-Mental State Examination (MMSE) recall are plotted with jitter to disperse points within groups.

We assessed the associations between each cognitive phenotype and age and BMI (Figure 1). The CANTAB-PAL score was significantly associated with age but not BMI as was the DLRT. Both the MMSE recall and verbal fluency significantly associated with both BMI and age.

**Figure 2:**
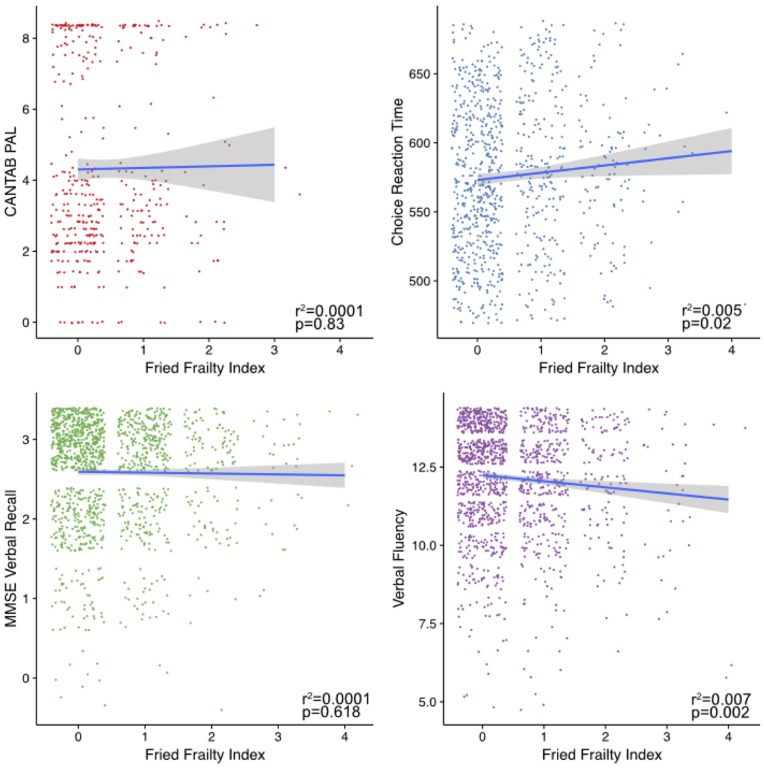
Frailty associations with cognitive phenotypes. Shown are plots of the cognitive measure scores against an individual’s frailty as measure by the Fried index. Fitted lines were made using the geom_smooth command in ggplot2 within R using the “lm” method.

Investigating the associations between the cognitive measures and frailty (Figure 2), only DLRT and verbal fluency were significantly associated.

### Cognitive Associations With Gut Microbiota

We carried out association analyses with gut microbiome traits, including four measures of alpha diversity, relative abundances of OTUs and taxonomic levels from genus through to phylum (in total, 416 microbiome traits; Supplementary Table S1). From these, we observed six FDR-significant associations after correcting for multiple testing (*p* < 0.05; Table 2). Three were negative associations between the DLRT and measures of alpha diversity including Chao1, phylogenetic diversity and OTU count. The other three significant associations were observed with verbal fluency. These were negative associations with the Shannon index and the Mollicutes class of bacteria, and a positive association with the class Bacilli. However, after adjustment for frailty using the Fried score, only associations between the DLRT and alpha diversity measures remained significant. In summary, the only significant observations after adjustment for frailty were lower microbiota alpha diversities associated with longer reaction times as measured by the DLRT.

**Table 2:** Significant associations observed between cognitive measures and the gut microbiome.

Cognitive test	Microbiome trait type	Microbiome trait	β-coefficient	*p*-value	FDR adjusted *q*-value	β-coefficient after frailty adjustment	*p*-value after frailty adjustment	FDR adjusted *p*-value after adjustment for frailty
DLRT	Alpha diversity	Chao1	−0.074	0.002	0.005	−0.073	0.002	0.01
DLRT	Alpha diversity	Phylogenetic diversity	−0.064	0.007	0.009	−0.058	0.015	0.02
DLRT	Alpha diversity	OTU count	−0.067	0.003	0.005	−0.062	0.005	0.0099
Inverted verbal fluency	Alpha Diversity	Shannon	−0.071	0.009	0.038	−0.062	0.023	0.09
Inverted verbal fluency	Class	Bacilli	0.073	0.007	0.044	0.062	0.02	0.087
Inverted verbal fluency	Class	Mollicutes	−0.074	0.007	0.044	−0.065	0.017	0.087

### Cognitive Associations With Gut Microbiota Considering PPI and Antibiotic Use and Diet

A range of factors, including medication use and diet, can influence the gut microbiome (Jackson et al., 2016b). We repeated the association analyses for all four cognitive measures, excluding individuals who reported taking PPIs or antibiotics and including a dietary index (the HEI) as a covariate. In these more limited subsets (verbal fluency *n* = 868, MMSE Recall *n* = 873, CANTAB PAL *n* = 274, DLRT *n* = 561) accounting for additional covariates, the previously observed associations between DLRT and alpha diversity measures again retained significance after adjusting for frailty (Table 3 and Supplementary Table S2). We also identified novel significant negative associations between DLRT and the order Burkholderiales and its class Betaproteobacteria.

**Table 3:** Significant associations between cognitive measures and the gut microbiome, when excluding individuals using antibiotics or proton pump inhibitors (PPIs) and including diet as a covariate.

Modeling type	Cognitive phenotype	Microbiome trait type	Microbiome trait	β-coefficient	*p*-value	FDR adjusted *p*-value	β-coefficient after adjustment for Frailty	*p*-value after adjustment for Frailty	FDR adjusted *p*-value after adjustment for frailty
Continuous	DLRT	Alpha diversity	Chao1	−0.079	0.006	0.013	−0.079	0.006	0.014
Continuous	DLRT	Alpha diversity	Phylogenetic diversity	−0.072	0.014	0.018	−0.067	0.021	0.028
Continuous	DLRT	Alpha diversity	OTU count	−0.076	0.004	0.013	−0.072	0.007	0.014
Continuous	DLRT	Class	Betaproteobacteria	−0.148	0.000	0.004	−0.138	0.001	0.009
Continuous	DLRT	Order	Burkholderiales	−0.147	0.000	0.005	−0.138	0.001	0.012

### Frailty Associations With Gut Microbiota

Modeling the association of frailty with all the microbiome traits and then including each of the four cognitive measures as continuous covariates independently in turn, we identified over 192 unique significant associations (Supplementary Table S3), covering a range of diversity, OTU and taxonomic measures. All but one of these associations remained significant when adjusting for the cognitive measures in turn. This was the association between frailty and Eubacterium that was non-significant after adjusting for verbal fluency. This highlights that association between frailty and the gut microbiota may be largely due to physical (as captured by the Fried index), as opposed to cognitive, aspects of frailty (Jackson et al., 2016c).

## Discussion

Here, we presented the first study to investigate the association of gut microbiota composition with cognitive measures in a large community dwelling aging cohort. We found choice reaction time had consistent negative associations with gut microbiota alpha diversity measures across all the models considered, which remained significant when considering host frailty, PPI and antibiotic use, and diet. These results are in concordance with a recent study of AD patients, who were shown to have a reduced gut microbiome diversity compared to healthy controls (Vogt et al., 2017). These also agree with numerous other studies of non-cognitive disorders that have found reduced alpha diversity associated with health deficits.

We observed a putative association with Burkholderiales order and DLRT that was dependent on the covariates used, and so should be viewed with caution. Of interest, one other study has identified Burkholderiales to differ in abundance in relation to neurological health, specifically Attention-deficit/Hyperactivity Disorder (ADHD) in comparison to a healthy control group (Akram, 2017). This may be relevant as cognitive impairment is found in ADHD, including (but not limited to) executive function and reaction time speed (Ivanchak et al., 2012; Sjöwall et al., 2013).

Association was seen between Verbal Fluency and Shannon diversity in the expected direction (higher fluency ability: higher microbiota diversity) however this was not significant after adjustment for the Fried score, suggesting confounding by physical frailty. Similarly, the associations observed between Verbal Fluency and *Bacilli* (lower relative abundance in cognitive deficit) and *Mollicutes* (lower in deficit) were only significant without considering host frailty. These results and the higher number of gut microbiota associations that we observed with frailty in comparison to cognitive measures (Supplementary Table S3), highlight the need for cognition-microbiome studies to incorporate physical health measures to separate the close links between cognitive and physical health. Muscle fitness has been shown to predict age-related changes in cognitive performance (Steves et al., 2016) and exercise interventions can improve cognition in older adults (Northey et al., 2018). The cognitive pathways that coordinate gait speed and grip strength include motor pathways and the motor cortex (Forssberg, 1999). However, we consider over-adjustment unlikely here as associations found with the cognitive task with the most reliance on motor function (DLRT) were not diminished by adjusting for frailty. The lesser effects observed with the other cognitive measures may also be due to reaction time being a more relevant measure of cognitive ability vis-à-vis the microbiome, further studies using alternate measures are warranted.

Diet, antibiotics and medication usage have shown to impact the microbiome (Jakobsson et al., 2010; Wu et al., 2011; Forslund et al., 2015; Jackson et al., 2016b). We found PPI, antibiotics and diet, as captured by the summary measure used here, were not responsible for the main association between reaction time and taxonomic diversity of the gut microbiota. Whilst these factors are major influences, they are not the only ones that could be considered. For instance, metformin, neuroleptic and opiate use has previously been associated with gut microbiota composition and there may be further medications and environmental influences that have not been considered (Flowers et al., 2017; Wu et al., 2017; Wang et al., 2018). Similarly, co-morbidities such as hypertension or type 2 diabetes that might be associated with both cognitive decline and gut microbiota composition (Zhang and Zhang, 2013; Pevsner-Fischer et al., 2017), and were not directly included in the models presented here. There is a strong correlation between Fried frailty and co-morbidity indexes however, both in our dataset (Livshits et al., 2018) and others (Rockwood et al., 2005). A comparison of two approaches to measuring frailty in elderly people, which means that most of the variance in microbiome attributable to co-morbidities is likely to be accounted for in the frailty adjusted models. Further studies, more tailored to address the questions of specific influences, will be required to probe the specificity of these associations. Additionally, bowel movements and stool consistency was not recorded within this study, and can influence gut microbiota richness, composition, enterotypes and bacterial growth rates (Tigchelaar et al., 2016; Vandeputte et al., 2016). Future studies should capture such measures, for instance using the Bristol Stool Chart, at the time of sample collection.

This study was also limited by the demographics of the twin cohort which, because of the previous research focus of the cohort, consists mainly of women, of middling socio-economic status and education typical of a volunteer group (Moayyeri et al., 2013; Steves et al., 2013). Correspondingly there may be a “healthy volunteer” bias. A small study in 39 men and 36 women showed differences in gut microbiota by gender, however these were highly influenced by BMI which we corrected for in our study. Nevertheless, it will beneficial to replicate our findings in a balanced cohort (Haro et al., 2016). The lack of associations with less sensitive cognitive measures such as the MMSE in our study could reflect the comparatively healthy nature of the cohort—alterations of gut microbiota composition may be enhanced in subjects with more severe alterations on neuropsychological tests or with an established diagnosis of mild cognitive impairment or dementia. However, positive findings in this study could indicate that gut microbiota is altered very early in the physiopathological process leading to cognitive impairment or dementia. Additionally, studies in more cognitively impaired groups could be more confounded by changes in physical health, diet and other lifestyle aspects as a consequence of impairment. Following individuals in longitudinal population-based studies and allowing cognitive decline to be quantified relative to individual’s baselines could in part address this. Longitudinal studies would also enable determination of the role of temporal variation in the gut microbiome and inference of the direction of causality between cognition-microbiome associations. Longitudinal cognitive aging and microbial assessments are planned within the TwinsUK cohort. Such future studies in this and other cohorts, would also benefit from the use of shotgun metagenomic sequencing, in place of 16S rRNA gene profiling as used here, which would uncover if gut microbiome functionality were associated with cognitive decline. In addition, measures of blood inflammatory markers could help disentangle the mechanisms that link gut microbiota with cognitive performance (Noble et al., 2017). Future studies could focus on obtaining potential inflammatory measures at similar time points of fecal collection.

In this study, we cannot determine whether cognitive ability and frailty are the cause, consequence or bystanders of differences in gut microbiota diversity and composition. However, the observed changes in the gut microbiome warrant further investigation as to whether they represent modifiable entities which could contribute to cognitive health. Should this be the case, maintenance or improvement of gut microbiota diversity could be a target to encourage cognitive health in aging, through dietary interventions to influence gut microbiota diversity (David et al., 2014; Carmody et al., 2015).

In conclusion, this is the first human study that links cognitive performance to gut microbiome in community dwelling ageing adults. Our research highlights the importance role of physical frailty when investigating cognitive performance. Although the causal direction between cognition and the composition of the gut microbiota remains unknown, we believe this study provides motivation for further investigations, and provides encouragement for translatable possibilities, such as dietary interventions aimed at ameliorating cognitive impairment in older adults.

## Data Availability

The 16S rRNA gene sequencing data used within these experiments is available as part of the European Nucleotide Archive (ENA) study with the accession number ERP015317. Phenotypic data are available for request from TwinsUK.

## Ethics Statement

This study was carried out in accordance with the recommendations of the National Health Service Health Research Authority by the London—Westminster Research Ethics Committee; REC ref: 12/LO/0227 (start 19/03/2012). The protocol was approved by the London—Westminster NRES Committee. All subjects gave written informed consent in accordance with the Declaration of Helsinki.

## Author Contributions

CS conceived and guided the study. MJ and MB carried out analysis of cognition and its associations with microbiota in TwinsUK. SV, JB, CS and TS were involved in coordination, collection, sequencing and processing of subjects and samples in the TwinsUK microbiota study, and provided relevant data and advised on analyses in this project. SV, MJ, MB and CS wrote the manuscript with input from all authors.

## Conflict of Interest Statement

TS is co-founder of MapMySelf Ltd and MapMyGut Ltd. The remaining authors declare that the research was conducted in the absence of any commercial or financial relationships that could be construed as a potential conflict of interest.

## Supplementary Material

TABLE S1 The results of association analyses vs. cognitive measures.

TABLE S2 Associations between cognitive measures and gut microbiota traits when excluding PPI and antibiotic users and including diet as a covariate.

TABLE S3 Significant results in association analyses vs. frailty.

## References

[B1] Akram H. (2017). Characterizing a link between gut microbiome and attention deficit hyperactive disorder. Honors College Research Collection Available online at: http://digitalcommons.fiu.edu/honors-research/4

[B2] Albert M. S. Jones K. Savage C. R. Berkman L. Seeman T. Blazer D.. (1995). Predictors of cognitive change in older persons: MacArthur studies of successful aging. Psychol. Aging 10, 578–589. 10.1037/0882-7974.10.4.578 8749585

[B3] American Psychiatric Association (2013). Diagnostic and Statistical Manual of Mental Disorders, Fifth Edition DSM 5. (Arlington, VA: American Psychiatric Association).

[B4] Auyeung T. W. Kwok T. Lee J. Leung P. C. Leung J. Woo J. (2008). Functional decline in cognitive impairment—the relationship between physical and cognitive function. Neuroepidemiology 31, 167–173. 10.1159/000154929 18784415 PMC2824577

[B5] Bajaj J. S. Ridlon J. M. Hylemon P. B. Thacker L. R. Heuman D. M. Smith S.. (2012). Linkage of gut microbiome with cognition in hepatic encephalopathy. Am. J. Physiol. Gastrointest. Liver Physiol. 302, G168–G175. 10.1152/ajpgi.00190.2011 21940902 PMC3345956

[B6] Baltes P. B. Lindenburger U. Staudinger U. M. (2006). “Life span theory in developmental psychology”, in Handbook of Child Psychology: Theoretical Models of Human Development eds Lerner R. M. Damon W. (Hoboken, NJ: John Wiley & Sons Inc.), 569–664.

[B7] Balzola F. Bernstein C. Ho G. T. (2011). A pyrosequencing study in twins shows that gastrointestinal microbial profiles vary with inflammatory bowel disease phenotypes: commentary. Inflamm. Bowel Dis. Monit. 11:166 10.1053/j.gastro.2010.08.049

[B8] Bates D. Mächler M. Bolker B. Walker S. (2015). Fitting linear mixed-effects models using lme4. J. Stat. Softw. 67:51 10.18637/jss.v067.i01

[B9] Benjamini Y. Hochberg Y. (1995). Controlling the false discovery rate: a practical and powerful approach to multiple testing. J. R. Stat. Soc. Ser. B 57, 289–300.

[B10] Bercik P. Denou E. Collins J. Jackson W. Lu J. Jury J.. (2011). The intestinal microbiota affect central levels of brain-derived neurotropic factor and behavior in mice. Gastroenterology 141, 599–609, 609.e1–609.e3. 10.1053/j.gastro.2011.04.052 21683077

[B11] Blackwell A. D. Sahakian B. J. Vesey R. Semple J. M. Robbins T. W. Hodges J. R. (2004). Detecting dementia: novel neuropsychological markers of preclinical Alzheimer’s disease. Dement. Geriatr. Cogn. Disord. 17, 42–48. 10.1159/000074081 14560064

[B12] Bowyer R. C. E. Jackson M. A. Pallister T. Skinner J. Spector T. D. Welch A. A.. (2018). Use of dietary indices to control for diet in human gut microbiota studies. Microbiome 6:77. 10.1186/s40168-018-0455-y 29695307 PMC5918560

[B13] Braniste V. Al-Asmakh M. Kowal C. Anuar F. Abbaspour A. Tóth M.. (2014). The gut microbiota influences blood-brain barrier permeability in mice. Sci. Transl. Med. 6:263ra158. 10.1126/scitranslmed.3009759 25411471 PMC4396848

[B14] Brayne C. (2007). The elephant in the room—healthy brains in later life, epidemiology and public health. Nat. Rev. Neurosci. 8, 233–239. 10.1038/nrn2091 17299455

[B15] Canevelli M. Cesari M. Remiddi F. Trebbastoni A. Quarata F. Vico C.. (2017). Promoting the assessment of frailty in the clinical approach to cognitive disorders. Front. Aging Neurosci. 9:36. 10.3389/fnagi.2017.00036 28286480 PMC5323399

[B16] Carabotti M. Scirocco A. Antonietta M. Severi C. (2015). The gut-brain axis: interactions between enteric microbiota, central and enteric nervous systems. Ann. Gastroenterol. 28, 203–209. 25830558 PMC4367209

[B17] Carmody R. N. Gerber G. K. Luevano J. M. Gatti D. M. Somes L. Svenson K. L.. (2015). Diet dominates host genotype in shaping the murine gut microbiota. Cell Host Microbe 17, 72–84. 10.1016/j.chom.2014.11.010 25532804 PMC4297240

[B18] Chen J. Chia N. Kalari K. R. Yao J. Z. Novotna M. Soldan M. M. P.. (2016). Multiple sclerosis patients have a distinct gut microbiota compared to healthy controls. Sci. Rep. 6:28484. 10.1038/srep28484 27346372 PMC4921909

[B19] Chupel M. U. Direito F. Furtado G. E. Minuzzi L. G. Pedrosa F. M. Colado J. C.. (2017). Strength training decreases inflammation and increases cognition and physical fitness in older women with cognitive impairment. Front. Physiol. 8:377. 10.3389/fphys.2017.00377 28659812 PMC5467003

[B20] Chyou P. H. White L. R. Yano K. Sharp D. S. Burchfiel C. M. Chen R.. (1996). Pulmonary function measures as predictors and correlates of cognitive functioning in later life. Am. J. Epidemiol. 143, 750–756. 10.1093/oxfordjournals.aje.a008812 8610684

[B21] Claesson M. J. Jeffery I. B. Conde S. Power S. E. O’Connor E. M. Cusack S.. (2012). Gut microbiota composition correlates with diet and health in the elderly. Nature 488, 178–184. 10.1038/nature11319 22797518

[B22] Cockrell F. M. Jr. Folstein M. F. (1988). Mini-mental state examination (MMSE). Psychopharmacol. Bull. 24, 689–692. 3249771

[B23] Coelho T. Paúl C. Gobbens R. J. J. Fernandes L. (2015). Determinants of frailty: the added value of assessing medication. Front. Aging Neurosci. 7:56. 10.3389/fnagi.2015.00056 25954195 PMC4404866

[B24] Comijs H. C. Dik M. G. Deeg D. J. H. Jonker C. (2004). The course of cognitive decline in older persons: results from the longitudinal aging study Amsterdam. Dement. Geriatr. Cogn. Disord. 17, 136–142. 10.1159/000076346 14739534

[B25] Cova I. Clerici F. Rossi A. Cucumo V. Ghiretti R. Maggiore L.. (2016). Weight loss predicts progression of mild cognitive impairment to Alzheimer’s disease. PLoS One 11:e0151710. 10.1371/journal.pone.0151710 26990757 PMC4798596

[B26] David L. A. Maurice C. F. Carmody R. N. Gootenberg D. B. Button J. E. Wolfe B. E.. (2014). Diet rapidly and reproducibly alters the human gut microbiome. Nature 505, 559–563. 10.1038/nature12820 24336217 PMC3957428

[B27] De Angelis M. Piccolo M. Vannini L. Siragusa S. De Giacomo A. Serrazzanetti D. I.. (2013). Fecal microbiota and metabolome of children with autism and pervasive developmental disorder not otherwise specified. PLoS One 8:e76993. 10.1371/journal.pone.0076993 24130822 PMC3793965

[B28] De Jager C. Blackwell A. D. Budge M. M. Sahakian B. J. (2005). Predicting cognitive decline in healthy older adults. Am. J. Geriatr. Psychiatry 13, 735–740. 10.1176/appi.ajgp.13.8.735 16085791

[B29] Deary I. J. Liewald D. Nissan J. (2011). A free, easy-to-use, computer-based simple and four-choice reaction time programme: the Deary-Liewald reaction time task. Behav. Res. Methods 43, 258–268. 10.3758/s13428-010-0024-1 21287123

[B30] Delaby C. Gabelle A. Blum D. Schraen-Maschke S. Moulinier A. Boulanghien J.. (2015). Central nervous system and peripheral inflammatory processes in Alzheimer’s disease: biomarker profiling approach. Front. Neurol. 6:181. 10.3389/fneur.2015.00181 26379616 PMC4547499

[B31] Dinan T. G. Cryan J. F. (2017). Brain-gut-microbiota axis-mood, metabolism and behaviour. Nat. Rev. Gastroenterol. Hepatol. 14, 69–70. 10.1038/nrgastro.2016.200 28053341

[B32] Edgar R. C. Haas B. J. Clemente J. C. Quince C. Knight R. (2011). UCHIME improves sensitivity and speed of chimera detection. Bioinformatics 27, 2194–2200. 10.1093/bioinformatics/btr381 21700674 PMC3150044

[B33] EPIC-Norfolk Nutritional Methods: Food Frequency Questionnaire (2014). University of Cambridge. Available online at: http://www.srl.cam.ac.uk/epic/nutmethod/FFQ.shtml [Accessed on 17 August 2014].

[B34] Finegold S. M. Molitoris D. Song Y. Liu C. Vaisanen M. Bolte E.. (2002). Gastrointestinal microflora studies in late-onset autism. Clin. Infect. Dis. 35, S6–S16. 10.1086/341914 12173102

[B35] Flowers S. A. Evans S. J. Ward K. M. McInnis M. G. Ellingrod V. L. (2017). Interaction between atypical antipsychotics and the gut microbiome in a bipolar disease cohort. Pharmacotherapy 37, 261–267. 10.1002/phar.1890 28035686

[B102] Forssberg H. (1999). Neural control of human motor development. Curr. Opin. Neurobiol. 9, 676–682. 10.1016/S0959-4388(99)00037-9 10607646

[B36] Forslund K. Hildebrand F. Nielsen T. Falony G. Le Chatelier E. Sunagawa S.. (2015). Disentangling type 2 diabetes and metformin treatment signatures in the human gut microbiota. Nature 528, 262–266. 10.1038/nature15766 26633628 PMC4681099

[B37] Fowler K. S. Saling M. M. Conway E. L. Semple J. M. Louis W. J. (1997). Computerized neuropsychological tests in the early detection of dementia: prospective findings. J. Int. Neuropsychol. Soc. 3, 139–146. 9126855

[B38] Fried L. P. Tangen C. M. Walston J. Newman A. B. Hirsch C. Gottdiener J.. (2001). Frailty in older adults: evidence for a phenotype. J. Gerontol. A Biol. Sci. Med. Sci. 56, M146–M156. 10.1093/gerona/56.3.M146 11253156

[B39] Goodrich J. K. Davenport E. R. Beaumont M. Jackson M. A. Knight R. Ober C.. (2016). Genetic determinants of the gut microbiome in UK twins. Cell Host Microbe 19, 731–743. 10.1016/j.chom.2016.04.017 27173935 PMC4915943

[B40] Goodrich J. K. Waters J. L. Poole A. C. Sutter J. L. Koren O. Blekhman R.. (2014). Human genetics shape the gut microbiome. Cell 159, 789–799. 10.1016/j.cell.2014.09.053 25417156 PMC4255478

[B41] Guenther P. M. Casavale K. O. Reedy J. Kirkpatrick S. I. Hiza H. A. B. Kuczynski K. J.. (2013). Update of the healthy eating index: HEI-2010. J. Acad. Nutr. Diet. 113, 569–580. 10.1016/j.jand.2012.12.016 23415502 PMC3810369

[B42] Haro C. Rangel-Zúñiga O. A. Alcalá-Díaz J. F. Gómez-Delgado F. Pérez-Martínez P. Delgado-Lista J.. (2016). Intestinal microbiota is influenced by gender and body mass index. PLoS One 11:e0154090. 10.1371/journal.pone.0154090 27228093 PMC4881937

[B43] Hsieh S. Schubert S. Hoon C. Mioshi E. Hodges J. R. (2013). Validation of the Addenbrooke’s cognitive examination III in frontotemporal dementia and Alzheimer’s disease. Dement. Geriatr. Cogn. Disord. 36, 242–250. 10.1159/000351671 23949210

[B44] Hugenholtz F. de Vos W. M. (2018). Mouse models for human intestinal microbiota research: a critical evaluation. Cell. Mol. Life Sci. 75, 149–160. 10.1007/s00018-017-2693-8 29124307 PMC5752736

[B101] Ivanchak N. Fletcher K. Jicha G. A. (2012). Attention-deficit/hyperactivity disorder in older adults: prevalence and possible connections to mild cognitive impairment. Curr. Psychiatry Rep. 14, 552–560. 10.1007/s11920-012-0305-8 22886581 PMC3718885

[B45] Jackson M. A. Bell J. T. Spector T. D. Steves C. J. (2016a). A heritability-based comparison of methods used to cluster 16S rRNA gene sequences into operational taxonomic units. PeerJ 4:e2341. 10.7717/peerj.2341 27635321 PMC5012273

[B46] Jackson M. A. Goodrich J. K. Maxan M. E. Freedberg D. E. Abrams J. A. Poole A. C.. (2016b). Proton pump inhibitors alter the composition of the gut microbiota. Gut 65, 749–756. 10.1136/gutjnl-2015-310861 26719299 PMC4853574

[B47] Jackson M. A. Jeffery I. B. Beaumont M. Bell J. T. Clark A. G. Ley R. E.. (2016c). Signatures of early frailty in the gut microbiota. Genome Med. 8:8. 10.1186/s13073-016-0262-7 26822992 PMC4731918

[B48] Jakobsson H. E. Jernberg C. Andersson A. F. Sjölund-Karlsson M. Jansson J. K. Engstrand L. (2010). Short-term antibiotic treatment has differing long-term impacts on the human throat and gut microbiome. PLoS One 5:e9836. 10.1371/journal.pone.0009836 20352091 PMC2844414

[B49] Jangi S. Gandhi R. Cox L. M. Li N. Von Glehn F. Yan R.. (2016). Alterations of the human gut microbiome in multiple sclerosis. Nat. Commun. 7:12015. 10.1038/ncomms12015 27352007 PMC4931233

[B50] Kang D. W. Park J. G. Ilhan Z. E. Wallstrom G. LaBaer J. Adams J. B.. (2013). Reduced incidence of prevotella and other fermenters in intestinal microflora of autistic children. PLoS One 8:e68322. 10.1371/journal.pone.0068322 23844187 PMC3700858

[B51] Khezrian M. Myint P. K. McNeil C. Murray A. D. (2017). A review of frailty syndrome and its physical, cognitive, and emotional domains in the elderly. Geriatrics 2:36 10.3390/geriatrics2040036 PMC6371193 31011046

[B52] Kluger A. Gianutsos J. G. Golomb J. Ferris S. H. George A. E. Franssen E.. (1997). Patterns of motor impairment in normal aging, mild cognitive decline, and early Alzheimer’ disease. J. Gerontol. B Psychol. Sci. Soc. Sci. 52B, P28–P39. 10.1093/geronb/52b.1.p28 9008673

[B53] Levy R. Borenstein E. (2014). Metagenomic systems biology and metabolic modeling of the human microbiome: from species composition to community assembly rules. Gut Microbes 5, 265–270. 10.4161/gmic.28261 24637600 PMC4063856

[B54] Livshits G. Lochlainn M. N. Malkin I. Bowyer R. Verdi S. Steves C. J.. (2018). Shared genetic influence on frailty and chronic widespread pain: a study from TwinsUK. Age Ageing 47, 119–125. 10.1093/ageing/afx122 28985290 PMC5860041

[B55] Miquel S. Leclerc M. Martin R. Chain F. Lenoir M. Raguideau S.. (2015). Identification of metabolic signatures linked to anti-inflammatory effects of Faecalibacterium prausnitzii. MBio 6:e00300-15. 10.1128/mBio.00300-15 25900655 PMC4453580

[B56] Moayyeri A. Hammond C. J. Hart D. J. Spector T. D. (2013). The UK adult twin registry (twinsUK resource). Twin Res. Hum. Genet. 16, 144–149. 10.1017/thg.2012.89 23088889 PMC3927054

[B57] Moos W. H. Faller D. V. Harpp D. N. Kanara I. Pernokas J. Powers W. R.. (2016). Microbiota and neurological disorders: a gut feeling. Biores. Open Access 5, 137–145. 10.1089/biores.2016.0010 27274912 PMC4892191

[B58] Noble E. E. Hsu T. M. Kanoski S. E. (2017). Gut to brain dysbiosis: mechanisms linking western diet consumption, the microbiome and cognitive impairment. Front. Behav. Neurosci. 11:9. 10.3389/fnbeh.2017.00009 28194099 PMC5277010

[B59] Northey J. M. Cherbuin N. Pumpa K. L. Smee D. J. Rattray B. (2018). Exercise interventions for cognitive function in adults older than 50: a systematic review with meta-analysis. Br. J. Sports Med. 52, 154–160. 10.1136/bjsports-2016-096587 28438770

[B60] Nyunt M. S. Z. Soh C. Y. Gao Q. Gwee X. Ling A. S. L. Lim W. S.. (2017). Characterisation of physical frailty and associated physical and functional impairments in mild cognitive impairment. Front. Med. 4:230. 10.3389/fmed.2017.00230 29326936 PMC5741611

[B61] Petersen C. Round J. L. (2014). Defining dysbiosis and its influence on host immunity and disease. Cell. Microbiol. 16, 1024–1033. 10.1111/cmi.12308 24798552 PMC4143175

[B62] Pevsner-Fischer M. Blacher E. Tatirovsky E. Ben-Dov I. Z. Elinav E. (2017). The gut microbiome and hypertension. Curr. Opin. Nephrol. Hypertens. 26, 1–8. 10.1097/mnh.0000000000000293 27798455

[B63] Puig K. L. Floden A. M. Adhikari R. Golovko M. Y. Combs C. K. (2012). Amyloid precursor protein and proinflammatory changes are regulated in brain and adipose tissue in a murine model of high fat diet-induced obesity. PLoS One 7:e30378. 10.1371/journal.pone.0030378 22276186 PMC3261903

[B103] R Core Team (2017). R: a language and environment for statistical computing. Avaliable online at: https://www.r-project.org/

[B64] Rockwood K. Song X. MacKnight C. Bergman H. Hogan D. B. McDowell I.. (2005). A global clinical measure of fitness and frailty in elderly people. CMAJ 173, 489–495. 10.1503/cmaj.050051 16129869 PMC1188185

[B65] Rogers G. B. Keating D. J. Young R. L. Wong M. L. Licinio J. Wesselingh S. (2016). From gut dysbiosis to altered brain function and mental illness: mechanisms and pathways. Mol. Psychiatry 21, 738–748. 10.1038/mp.2016.50 27090305 PMC4879184

[B66] Sartori A. C. Vance D. E. Slater L. Z. Crowe M. (2012). The impact of inflammation on cognitive function in older adults: implications for healthcare practice and research. J. Neurosci. Nurs. 44, 206–217. 10.1097/jnn.0b013e3182527690 22743812 PMC3390758

[B67] Schaie K. W. Willis S. L. Caskie G. I. L. (2004). The Seattle longitudinal study: relationship between personality and cognition. Neuropsychol. Dev. Cogn. B Aging Neuropsychol. Cogn. 11, 304–324. 10.1080/13825580490511134 16755303 PMC1474018

[B68] Scheperjans F. Aho V. Pereira P. A. B. Koskinen K. Paulin L. Pekkonen E.. (2015). Gut microbiota are related to Parkinson’s disease and clinical phenotype. Mov. Disord. 30, 350–358. 10.1002/mds.26069 25476529

[B69] Searle S. D. Rockwood K. (2015). Frailty and the risk of cognitive impairment. Alzheimers Res. Ther. 7:54. 10.1186/s13195-015-0140-3 26240611 PMC4523015

[B70] Segain J. P. Raingeard de la Blétière D. Bourreille A. Leray V. Gervois N. Rosales C.. (2000). Butyrate inhibits inflammatory responses through NFkappaB inhibition: implications for Crohn’s disease. Gut 47, 397–403. 10.1136/gut.47.3.397 10940278 PMC1728045

[B71] Sjöwall D. Roth L. Lindqvist S. Thorell L. B. (2013). Multiple deficits in ADHD: executive dysfunction, delay aversion, reaction time variability, and emotional deficits. J. Child Psychol. Psychiatry 54, 619–627. 10.1111/jcpp.12006 23061803 PMC3758957

[B72] Sokol H. Pigneur B. Watterlot L. Lakhdari O. Bermúdez-Humarán L. G. Gratadoux J.-J.. (2008). Faecalibacterium prausnitzii is an anti-inflammatory commensal bacterium identified by gut microbiota analysis of Crohn disease patients. Proc. Natl. Acad. Sci. U S A 105, 16731–16736. 10.1073/pnas.0804812105 18936492 PMC2575488

[B73] Steves C. J. Jackson S. H. D. Spector T. D. (2013). Cognitive change in older women using a computerised battery: a longitudinal quantitative genetic twin study. Behav. Genet. 43, 468–479. 10.1007/s10519-013-9612-z 23990175 PMC3825151

[B74] Steves C. J. Mehta M. M. Jackson S. H. D. Spector T. D. (2016). Kicking back cognitive ageing: leg power predicts cognitive ageing after ten years in older female twins. Gerontology 62, 138–149. 10.1159/000441029 26551663 PMC4789972

[B75] Stilling R. M. Dinan T. G. Cryan J. F. (2014). Microbial genes, brain and behaviour-epigenetic regulation of the gut-brain axis. Genes Brain Behav. 13, 69–86. 10.1111/gbb.12109 24286462

[B76] Tigchelaar E. F. Bonder M. J. Jankipersadsing S. A. Fu J. Wijmenga C. Zhernakova A. (2016). Gut microbiota composition associated with stool consistency. Gut 65, 540–542. 10.1136/gutjnl-2015-310328 26276682

[B77] Tombaugh T. N. McIntyre N. J. (1992). The mini-mental state examination: a comprehensive review. J. Am. Geriatr. Soc. 40, 922–935. 10.1111/j.1532-5415.1992.tb01992.x 1512391

[B78] van Tongeren S. P. Slaets J. P. J. Harmsen H. J. M. Welling G. W. (2005). Fecal microbiota composition and frailty. Appl. Environ. Microbiol. 71, 6438–6442. 10.1128/aem.71.10.6438-6442.2005 16204576 PMC1265947

[B79] Vandeputte D. Falony G. Vieira-Silva S. Tito R. Y. Joossens M. Raes J. (2016). Stool consistency is strongly associated with gut microbiota richness and composition, enterotypes and bacterial growth rates. Gut 65, 57–62. 10.1136/gutjnl-2015-309618 26069274 PMC4717365

[B80] Vida C. Martinez de Toda I. Garrido A. Carro E. Molina J. A. De la Fuente M. (2018). Impairment of several immune functions and redox state in blood cells of Alzheimer’s disease patients. Relevant role of neutrophils in oxidative stress. Front. Immunol. 8:1974. 10.3389/fimmu.2017.01974 29375582 PMC5768621

[B81] Vogt N. M. Kerby R. L. Dill-McFarland K. A. Harding S. J. Merluzzi A. P. Johnson S. C.. (2017). Gut microbiome alterations in Alzheimer’s disease. Sci. Rep. 7:13537. 10.1038/s41598-017-13601-y 29051531 PMC5648830

[B82] Wang F. Meng J. Zhang L. Johnson T. Chen C. Roy S. (2018). Morphine induces changes in the gut microbiome and metabolome in a morphine dependence model. Sci. Rep. 8:3596. 10.1038/s41598-018-21915-8 29483538 PMC5827657

[B83] Wong A. L. Haith A. M. Krakauer J. W. (2015). Motor planning. Neuroscieiist 21, 385–398. 10.1177/1073858414541484 24981338

[B84] Woods A. J. Cohen R. A. Pahor M. (2013). Cognitive frailty: frontiers and challenges. J. Nutr. Health Aging 17, 741–743. 10.1007/s12603-013-0398-8 24154645 PMC4471842

[B85] Wu G. D. Chen J. Hoffmann C. Bittinger K. Chen Y. Y. Keilbaugh S. A.. (2011). Linking long-term dietary patterns with gut microbial enterotypes. Science 334, 105–108. 10.1126/science.1208344 21885731 PMC3368382

[B86] Wu H. Esteve E. Tremaroli V. Khan M. T. Caesar R. Mannerås-Holm L.. (2017). Metformin alters the gut microbiome of individuals with treatment-naive type 2 diabetes, contributing to the therapeutic effects of the drug. Nat. Med. 23, 850–858. 10.1038/nm.4345 28530702

[B87] Yu R. Morley J. E. Kwok T. Leung J. Cheung O. Woo J. (2018). The effects of combinations of cognitive impairment and pre-frailty on adverse outcomes from a prospective community-based cohort study of older chinese people. Front. Med. 5:50. 10.3389/fmed.2018.00050 29600247 PMC5863513

[B88] Zhang Y. Zhang H. (2013). Microbiota associated with type 2 diabetes and its related complications. Food Sci. Hum. Wellness 2, 167–172. 10.1016/j.fshw.2013.09.002

